
JOURNAL INFORMATION
==============================
NLM Title Abbreviation: Rheumatology (Oxford)
Journal ID: Rheumatology (Oxford)
NLM Journal ID: 100883501
Journal ID: brheum

Rheumatology (Oxford, England)

ISSN: 1462-0324
EISSN: 1462-0332

ARTICLE INFORMATION
==============================
PMCID: PMC7989159
PMID: 33667295
DOI: 10.1093/rheumatology/keab201
Article ID: keab201
Article version: 1
Subjects: Letter to the Editor (Other), AcademicSubjects/MED00360

The impact of telerheumatology and COVID-19 on outcomes in a tertiary rheumatology service: a retrospective audit

http://orcid.org/0000-0002-1333-2384 Zhu Wendy 1
De Silva Thilinie 2
Eades Laura 2
Morton Susan 2
Ayoub Sally 1 2
Morand Eric 1 2
Antony Anna 1 2
1 School of Clinical Sciences, Monash University , Melbourne, Australia
2 Department of Rheumatology, Monash Health , Melbourne, Australia
Corresponding author: Wendy Zhu, 246 Clayton Road, Clayton VIC Australia 3168, Wendy.zhu@monashhealth.org
Electronic publication date: 2021 Mar 1
Electronic Location ID: keab201
Received 2021 Feb 8; Revised 2021 Feb 22; Accepted 2021 Feb 23
Copyright: © The Author(s) 2021. Published by Oxford University Press on behalf of the British Society for Rheumatology.
Copyright year: 2021
License: This is an Open Access article distributed under the terms of the Creative Commons Attribution License (http://creativecommons.org/licenses/by/4.0/), which permits unrestricted reuse, distribution, and reproduction in any medium, provided the original work is properly cited.
License URL: https://creativecommons.org/licenses/by/4.0/

Page count: 7
edited-state: accepted-manuscript
article-lifecycle: PAP

==============================
**The impact of telerheumatology and COVID-19 on outcomes in a tertiary rheumatology service: a retrospective audit**

Wendy Zhu[1], Thilinie De Silva[2], Laura Eades[2], Susan Morton[2], Sally Ayoub[1,2] , Eric Morand[1,2], Anna Antony[1,2]

1 School of Clinical Sciences, Monash University, Melbourne, Australia.

2 Department of Rheumatology, Monash Health, Melbourne, Australia

**Corresponding author:**

Wendy Zhu

246 Clayton Road, Clayton VIC Australia 3168

Wendy.zhu@monashhealth.org

ORCiD iD: https://orcid.org/0000-0002-1333-2384

© The Author(s) 2021. Published by Oxford University Press on behalf of the British Society for Rheumatology. All rights reserved. For permissions, please email: journals.permissions@oup.com

Dear Editor,

Recent studies suggest that telerheumatology has been feasible and acceptable to patients during the COVID-19 pandemic (1-3), however, the impact it has on patient care is poorly understood. We conducted a retrospective audit to evaluate the impact of telerheumatology and the pandemic on the care of rheumatology patients in a tertiary outpatient service in 2020.

Monash Health is the largest public health service provider in the state of Victoria, providing healthcare to 25% of the population in the socioeconomically and culturally diverse city of Melbourne, Australia. Electronic records from all rheumatology clinics (general rheumatology, Rheumatoid Arthritis, Systemic Lupus Erythematosus, Vasculitis, Scleroderma, Fibromyalgia and complex rheumatology) were reviewed. Two study periods were included: a comparator historical cohort (April to May 2019), and the COVID-19 telerheumatology cohort (April to May 2020). Mann-Whitney U and Chi-square tests were used to assess for differences in baseline variables between cohorts. Univariate analysis was used to estimate odds ratios (ORs) for binary outcomes, while the Mann-Whitney U test was used for continuous outcomes. Univariate analysis was also used to assess the impact of baseline variables on the odds of requiring a subsequent face-to-face (F2F) review following telerheumatology consultation in 2020. Statistical analyses were undertaken using SPSS v23.0 (IBM Corp., Armonk, NY, USA). Ethics approval was obtained from Monash Health (Ref: RES-20-0000-623Q-68097).

3,040 appointments were identified in the study periods: 1,443 from 2019 and 1,597 from 2020. There was no statistically significant difference in the age, sex, proportion of new/review appointments, or frequency of immunosuppression use between the cohorts. (Supplementary Table 1). The median age of the 2020 cohort was 54 years and there was a higher proportion of females (69.8%). Inflammatory arthritis (IA) was the most common diagnosis (35.1%), followed by lupus/connective tissue disease (CTD) (25.3%) and vasculitis

(8.4%). IA was a more common diagnosis in 2020 (35.1% vs 31%, p=0.024) which may reflect the prioritisation of urgent referrals and appointment allocation during the pandemic. In 2020, 96.7% of appointments (n=1,444) were conducted via telerheumatology, almost exclusively via telephone.

In patients without an existing rheumatological diagnosis, the odds of making a diagnosis were significantly lower in 2020 (28.6% vs 57.4%; OR 0.30 [95% CI: 0.16-0.53] p<0.001). This supports, in a much larger sample, previous literature indicating that diagnostic confidence and accuracy with telephone-based telerheumatology is lower than with F2F appointments (4-6).

Clinicians were less likely to change immunosuppressive therapy in 2020 (22.6% vs 27.4%; OR 0.78 [95% CI 0.65-0.92] p=0.004). This was mostly driven by less de-escalation in therapy (10% vs 12.6%; OR 0.75 [95% CI 0.59-0.95]; p=0.019) (Table 1). This may suggest a preference to minimise the risk of flares and avoid rescue immunosuppression, or the inability to confidently assess disease activity via telerheumatology. Formalised patient self-assessment in our cohort was only utilised for patients on biological or targeted synthetic disease-modifying anti-rheumatic drugs to meet the requirements of the Pharmaceutical Benefits Scheme. However, our findings also indicate that clinicians were comfortable increasing or switching therapy in 2020 despite the vast majority of appointments being conducted via telerheumatology.

Appointment attendance increased in 2020, with non-attendance falling to 6.5% from 10.9% in 2019 (OR 0.57 [95% CI: 0.44-0.74]; p<0.001). The odds of discharging a patient were lower in 2020 (3.9% vs 6%; OR 0.64 [95% CI 0.46-0.89]; p=0.008). This statistical significance was attenuated when patients who failed to attend were excluded, indicating that clinicians were less likely to discharge patients who failed to attend appointments during the pandemic. Unplanned hospital presentations and planned admissions were reduced in 2020

($p<0.05$), mirroring lower presentation rates to Australian emergency departments during the pandemic and in keeping with hospital attendance hesitancy due to concerns about hospital-acquired transmission of COVID-19. (7). However, the percentage of patients with unplanned rheumatological hospital presentations was unchanged.

Patients seen in 2020 also required earlier follow-up appointments ($p<0.001$), which may relate to the inability to conduct a physical examination (Supplementary Figure 1). Amongst patients seen via telerheumatology in 2020 (n=1,444), a subsequent and additional F2F appointment was required in 9.4%. Predictors of needing a F2F review were being a new patient (OR 6.28 [95% CI: 4.10-9.64]; $p<0.001$), not having a rheumatological diagnosis (OR 18.43 [95% CI: 2.35-144.63]; p=0.006), or having a diagnosis of IA (OR 2.85 [95% CI: 1.40-5.80]; p=0.004) or lupus/CTD (OR 3.22 [95% CI: 1.11-9.32]; p=0.031).

In summary, most patients in 2020 were seen via telerheumatology. Telerheumatology use during the COVID-19 pandemic was associated with improved appointment attendance, but with diagnostic delay, reduced likelihood of changing existing immunosuppressive therapy, earlier requirement for review, and lower likelihood of discharge. While the effects of telerheumatology cannot be differentiated from changes in practice related to the pandemic, these findings suggest telephone-based telerheumatology may have a negative impact on the timeliness of management of rheumatology patients.

**Rheumatology key message:** Telephone-based telerheumatology may affect the timeliness of management of rheumatology patients.

**Table 1: Outcomes of rheumatology appointments in 2019 and 2020**

| | **2019** n=1286 | **2020** n=1493 | **Odds Ratio [95% CI]** | **p-value** |
|---|---|---|---|---|
| **Ability to make a diagnosis for those without a rheumatological diagnosis % (n)** | 57.4 (54/94) | 28.6 (30/105) | 0.30 [0.164-0.534] | **<0.001** |
| **Change in immunosuppression % (n)** | 27.4 (352) | 22.6 (338) | 0.78 [0.654-0.923] | **0.004** |
| **Type of change in immunosuppression % (n)** | | | | |
| No change | 72.9 (938) | 77.4 (1155) | REFERENCE | |
| Escalated | 12.9 (166) | 11.7 (174) | 0.85 [0.677-1.071] | 0.169 |
| De-escalated | 12.6 (162) | 10.0 (150) | 0.75 [0.593-0.954] | **0.019** |
| Switch | 1.6 (20) | 0.9 (14) | 0.57 [0.286-1.132] | 0.108 |
| **Next planned review (months) Median [IQR]** | 3.0 [2.00-4.00] | 3.0 [2.00-4.00] | - | **<0.001** |

| | | | | |
|---|---|---|---|---|
| **Did Not Attend (DNA) % (n)** | 10.9 (157/1443) | 6.5 (104/1597) | 0.57 [0.440-0.739] | **<0.001** |
| **Discharged % (n)** | 6 (87/1443) | 3.9 (63/1597) | 0.64 [0.459-0.892] | **0.008** |
| **Follow-up Phone Call Required % (n)** | 2.3 (29) | 3.2 (48) | 1.44 [0.901-2.293] | 0.127 |
| **Changes in analgesia % (n)** | 7.5 (96) | 5.3 (79) | 0.69 [0.509-0.942] | **0.019** |
| **Injection or Aspirate performed % (n)** | 2.3 (29) | 0.4 (6) | 0.18 [0.072-0.423] | **<0.001** |
| **Unplanned hospital presentation % (n)** | 7.3 (94) | 5.4 (80) | 0.72 [0.528-0977] | **0.034** |
| **Unplanned rheumatological hospital presentation % (n)** | 44.7 (42) | 35 (28) | 0.67 [0.361-1.231] | 0.194 |
| **Unplanned admission % (n)** | 4.1 (53) | 2.6 (39) | 0.62 [0.410-0.950] | **0.027** |
| **Planned Admission or Procedure % (n)** | 2.6 (33) | 1.0 (15) | 0.38 [0.208-0.710] | **0.002** |

### References

1. Costa L, Tasso M, Scotti N, Mostacciuolo E, Girolimetto N, Foglia F, et al. Telerheumatology in COVID-19 era: a study from a psoriatic arthritis cohort. Annals of the Rheumatic Diseases. 2020.
2. Zhang Y, Wang J, Zhao L, Xiao J, Shi Z. Online management of rheumatoid arthritis during COVID-19 pandemic. Annals of the Rheumatic Diseases. 2020.
3. Antony A, Connelly K, De Silva T, Eades L, Tillett W, Ayoub S, et al. Perspectives of patients with rheumatic diseases in the early phase of COVID-19. Arthritis Care & Research. 2020.
4. Nguyen-Oghalai TU, Hunter K, Lyon M. Telerheumatology: The VA Experience. Southern medical journal. 2018;111(6):359-62.
5. Leggett P, Graham L, Steele K, Gilliland A, Stevenson M, O'Reilly D, et al. Telerheumatology--diagnostic accuracy and acceptability to patient, specialist, and general practitioner. British Journal of General Practice. 2001;51(470):746-8.
6. Cervera R, Khamashta MA, Font J, Sebastiani GD, Gil A, Lavilla P, et al. Morbidity and mortality in systemic lupus erythematosus during a 10-year period: a comparison of early and late manifestations in a cohort of 1,000 patients. Medicine. 2003;82(5):299-308.
7. Kam AW, Chaudhry SG, Gunasekaran N, White AJR, Vukasovic M, Fung AT. Fewer presentations to metropolitan emergency departments during the COVID-19 pandemic. Medical Journal of Australia. 2020;213(8):370-1.

### Contributorship Statement:

WZ and AA contributed to the study conception, design, data collection, data interpretation, and drafting of the manuscript. AA contributed to the statistical analysis. All authors reviewed the manuscript and gave final approval of this version for publication.

### Data Statement:

Data are available upon request.

### Funding Statement:

No specific funding was received from any bodies in the public, commercial, or not-for-profit sectors to carry out the work described in this article.

### Conflicts of Interest:

The authors declare no conflicts of interest.
